# Effects of two different regional citrate anticoagulation CVVH protocols on acid-base status and phosphate supplementation

**DOI:** 10.1186/cc12375

**Published:** 2013-03-19

**Authors:** S Morabito, V Pistolesi, V Pistolesi, L Tritapepe, L Tritapepe, E Vitaliano, E Vitaliano, L Zeppilli, L Zeppilli, F Polistena, F Polistena, E Fiaccadori, E Fiaccadori, A Pierucci, A Pierucci

**Affiliations:** 1 Umberto I, Policlinico di Roma, 'Sapienza' University, Rome, Italy; 2 Umberto I, Policlinico di Roma, Rome, Italy; 3 Pertini Hospital, Rome, Italy; 4 University of Parma Medical School, Parma, Italy

## Introduction

Regional citrate anticoagulation (RCA) is increasingly used in high bleeding risk patients undergoing CRRT. Regardless of anticoagulation protocol, hypophosphatemia occurs frequently in CRRT. The aim was to evaluate the effects on electrolyte and acid-base status of a new RCA-CVVH protocol using an 18 mmol/l citrate solution combined with a phosphate-containing replacement fluid, compared with a previously adopted RCA-CVVH protocol combining a 12 mmol/l citrate solution with a conventional replacement fluid.

## Methods

Until September 2011, RCA-CVVH was routinely performed in our centre with a 12 mmol/l citrate solution and a postdilution replacement fluid with bicarbonate (HCO_3_^- ^32, Ca^2+ ^1.75, Mg^2+ ^0.5, K^+ ^2 mmol/l) (protocol A). In cases of metabolic acidosis, not related to inappropriate citrate metabolism and persisting after optimization of RCA-CVVH parameter setting, bicarbonate infusion was scheduled. Starting from September 2011, in order to optimize buffer balance and to reduce the need for phosphate supplementation, a new RCA-CVVH protocol has been designed using an 18 mmol/l citrate solution combined with a recently introduced phosphate-containing replacement fluid with bicarbonate (HCO_3_^- ^30, phosphate 1.2, Ca^2+ ^1.25, Mg^2+ ^0.6, K^+ ^4 mmol/l) (protocol B).

## Results

In 30 cardiac surgery patients with AKI, acid-base status and electrolytes have been evaluated comparing protocol A (20 patients, running time 5,283 hours) versus protocol B (10 patients, 1,170 hours) (median (IQR)): pH 7.40 (7.36 to 7.44) versus 7.43 (7.41 to 7.47) (*P *0.0001), bicarbonate 22.1 (20.9 to 23.5) versus 24.4 (23.2 to 25.6) mmol/l (*P *0.0001), base excess -3.1 (-4.6 to -1.15) versus 0 (-1.5 to 1.1) (*P *0.0001), systemic Ca^2+ ^1.16 (1.1 to 1.23) versus 1.14 (1.08 to 1.19) mmol/l (*P *0.0001), phosphate 0.7 (0.5 to 1) versus 1.1 (0.9 to 1.4) mmol/l (*P *0.0001). Protocol A required bicarbonate infusion in 90% of patients (6 ± 6.4 mmol/hour) and sodium phosphate supplementation in all cases (4.1 ±3 2.4 g/day). A lower amount of phosphate supplementation (0.9 ± 23 g/day) was needed in 30% of patients undergoing protocol B while bicarbonate infusion was never required. Filter life was comparable (51.8 ± 36.5 vs. 46.8 ± 30.3 hours, *P *= NS).

## Conclusion

Protocol B provided a buffer balance more positive than protocol A and allowed one to adequately control acid-base status without additional bicarbonate infusion and in the absence of alkalosis, despite the use of a standard bicarbonate concentration replacement solution. Furthermore, the combination of a phosphate-containing replacement fluid appeared effective to prevent hypophosphatemia.

